# Synergistic cytotoxic effects of bortezomib and CK2 inhibitor CX-4945 in acute lymphoblastic leukemia: turning off the prosurvival ER chaperone BIP/Grp78 and turning on the pro-apoptotic NF-κB

**DOI:** 10.18632/oncotarget.6361

**Published:** 2015-11-22

**Authors:** Francesca Buontempo, Ester Orsini, Annalisa Lonetti, Alessandra Cappellini, Francesca Chiarini, Camilla Evangelisti, Cecilia Evangelisti, Fraia Melchionda, Andrea Pession, Alice Bertaina, Franco Locatelli, Jessika Bertacchini, Luca Maria Neri, James A. McCubrey, Alberto Maria Martelli

**Affiliations:** ^1^ Department of Biomedical and Neuromotor Sciences, University of Bologna, Bologna, Italy; ^2^ Pediatric Oncology and Hematology Unit “Lalla Seràgnoli”, S. Orsola-Malpighi Hospital, University of Bologna, Bologna, Italy; ^3^ Department of Human Social and Health Sciences, Campus Folcara, University of Cassino, Cassino, Italy; ^4^ Muscoloskeletal Cell Biology Laboratory, IOR, Bologna, Italy; ^5^ Institute of Molecular Genetics, National Research Council-Rizzoli Orthopedic Institute, Bologna, Italy; ^6^ Department of Pediatric Hematology and Oncology, IRCCS Bambino Gesù Children's Hospital, Rome, Italy; ^7^ Department of Surgery, Medicine, Odontostomatology and Morphological Sciences, University of Modena, Modena, Italy; ^8^ Department of Morphology, Surgery and Experimental Medicine, University of Ferrara, Ferrara, Italy; ^9^ Department of Microbiology and Immunology, School of Medicine, East Carolina University, Greenville, NC, USA

**Keywords:** acute lymphoblastic leukemia, CK2, unfolded protein response, BIP/Grp78, NF-κB

## Abstract

The proteasome inhibitor bortezomib is a new targeted treatment option for refractory or relapsed acute lymphoblastic leukemia (ALL) patients. However, a limited efficacy of bortezomib alone has been reported. A terminal pro-apoptotic endoplasmic reticulum (ER) stress/unfolded protein response (UPR) is one of the several mechanisms of bortezomib-induced apoptosis. Recently, it has been documented that UPR disruption could be considered a selective anti-leukemia therapy. CX-4945, a potent casein kinase (CK) 2 inhibitor, has been found to induce apoptotic cell death in T-ALL preclinical models, via perturbation of ER/UPR pathway. In this study, we analyzed in T- and B-ALL preclinical settings, the molecular mechanisms of synergistic apoptotic effects observed after bortezomib/CX-4945 combined treatment. We demonstrated that, adding CX-4945 after bortezomib treatment, prevented leukemic cells from engaging a functional UPR in order to buffer the bortezomib-mediated proteotoxic stress in ER lumen. We documented that the combined treatment decreased pro-survival ER chaperon BIP/Grp78 expression, via reduction of chaperoning activity of Hsp90. Bortezomib/CX-4945 treatment inhibited NF-κB signaling in T-ALL cell lines and primary cells from T-ALL patients, but, intriguingly, in B-ALL cells the drug combination activated NF-κB p65 pro-apoptotic functions. In fact in B-cells, the combined treatment induced p65-HDAC1 association with consequent repression of the anti-apoptotic target genes, Bcl-xL and XIAP. Exposure to NEMO (IKKγ)-binding domain inhibitor peptide reduced the cytotoxic effects of bortezomib/CX-4945 treatment. Overall, our findings demonstrated that CK2 inhibition could be useful in combination with bortezomib as a novel therapeutic strategy in both T- and B-ALL.

## INTRODUCTION

Acute lymphoblastic leukemia (ALL) is a malignant disorder that originates from hematopoietic precursors committed to either T- or B-cell lineage. T-ALL comprises 15-20% of ALL cases, whereas B-ALL represents 80-85% [1]. Overall, ALL is the most common pediatric malignancy [2]. ALL survival rates have improved especially in children, due to the recognition of the biologic heterogeneity of ALL, the utilization of risk-adapted therapy, and the development of protocols with optimized chemotherapy combinations. However, relapsed ALL is difficult to treat [3]. Therapy for relapsed pediatric ALL is hindered by low remission rates and high toxicity, especially in second and subsequent relapses within three years of diagnosis [4-6]. Therapeutic alternatives with effective anti-leukemic potential and lower toxicity profile are needed for these extensively pretreated patients. Currently, several new treatment options for relapsed ALL have emerged, including deoxyadenosine analogs (clofarabine and nelarabine), monoclonal antibodies (alemtuzumab and epratuzumab), and the proteasome inhibitor, bortezomib [3, 7].

Bortezomib (Velcade, formerly PS-341) is the first proteasome inhibitor approved by the U.S. FDA for multiple myeloma (MM) [8, 9], and, more recently, for relapsed mantle cell lymphoma (MCL) treatment [10]. Bortezomib is a dipeptidyl boronic acid analog that reversibly inhibits the 26S proteasome. The proteasome has been successfully targeted for the treatment of MM and MCL but, in other hematologic malignancies, bortezomib has been less effective as a single agent. However, phase I studies of Children's Oncology Group and Pediatric Preclinical Testing Program have demonstrated that, in refractory and/or relapsed ALL, bortezomib could act as a chemosensitizing agent [11-13]. In particular, the Therapeutic Advances in Childhood Leukemia & Lymphoma (TACL) phase I and II studies have documented that bortezomib is able to restore sensitivity of leukemic cells to conventional dexamethasone, asparaginase, vincristine, doxorubicin, and cytarabine chemotherapy [14]. Moreover, about twenty therapeutic targeted small molecules are currently being assessed in combination with bortezomib in more than 40 ongoing early-phase clinical trials. The majority of the studies are evaluating patients with hematologic malignancies and have demonstrated that bortezomib displays an excellent synergistic activity with deacetylase inhibitors [15], kinase inhibitors [16, 17], and endoplasmic reticulum (ER) stress modulators [18, 19].

Bortezomib molecular mechanisms of action in ALL are still not completely understood. Previous reports have highlighted that, in most cases, it could affect activation of the NF-κB pathway through IκBα stabilization [20, 21], but it also up-regulated NF-κB activity [22]. In MM cells, it has been shown that a terminal pro-apoptotic ER stress/UPR elicitation is involved in bortezomib-induced cytotoxicity [23]. Interestingly, ER stress/UPR signaling is recently gaining increasing recognition as a key targetable pathway in ALL [24-27].

CX-4945 is a potent and selective small molecule inhibitor of the casein kinase 2 (CK2), currently used in phase I clinical trial in patients with advanced solid cancers (NCT00891280) and in MM patients (NCT01199718). CK2 is a pivotal pro-survival protein kinase that creates a cellular environment positive to neoplastic cells by phosphorylating an impressive number of substrates [28]. CK2 targets IKK/NF-κB signaling [28-30], the Akt pathway [31], and the ER stress/UPR axis [26, 32, 33]. We have recently demonstrated that CK2 inhibition by CX-4945 potently down-regulated UPR signaling in T-ALL preclinical models [26]. Recently, the efficacy of a bortezomib/CX-4945 combination has been described in a study on MM and MCL cells [34]. In the present work, we explored the synergistic cytotoxic effects of a bortezomib/CX-4945 combination in a panel of T- and B-ALL cell lines and lymphoblasts from T- and B-ALL patients. Our starting hypothesis was that adding CX-4945 to leukemic cells previously exposed to bortezomib, could prevent cancer cells from engaging the UPR in order to effectively buffer the increased proteotoxic load in the ER lumen, thus enhancing sensitivity to the proteasome inhibitor. Bortezomib-induced apoptotic effects were significantly amplified by CK2 inhibition and apoptotic cell death was accompanied by down-regulation of ER stress/UPR signaling. Exposure to combined treatment provoked a reduction of pro-survival ER chaperon BIP/Grp78 expression, the main activator of the UPR and a well-known facilitator of tumor cell evasion from chemotherapeutics. We also demonstrated that modulation of NF-κB pathway was involved in bortezomib/CX-4945 mechanisms of action. However, in B-ALL cell lines and patient lymphoblasts, the drug combination was able to unleash NF-κB pro-apoptotic ability. Overall, our findings provided a rationale for the use of CK2 inhibitors in bortezomib-based combination protocols in ALL.

## RESULTS

### A bortezomib/CX-4945 combined treatment induces synergistic apoptotic effects in T- and B-ALL cell lines

**Figure 1:**
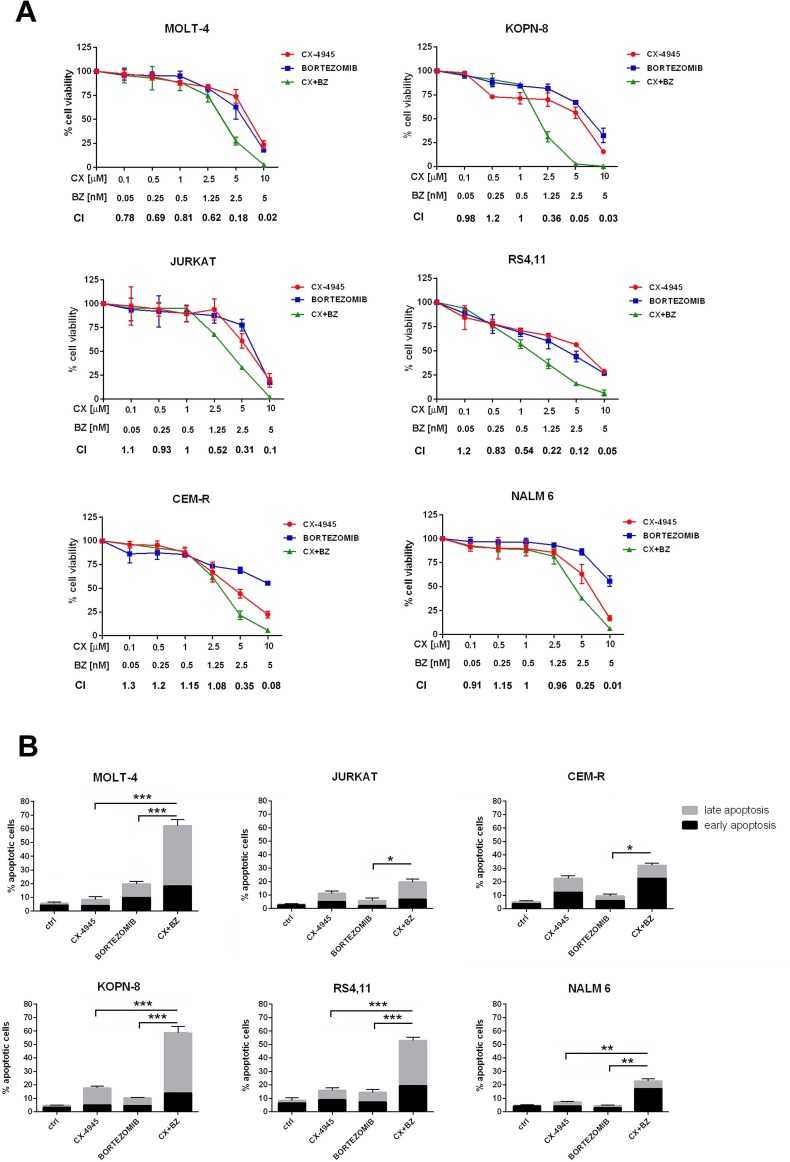
Bortezomib/CX-4945 combination induces synergistic apoptotic effects in T- and B-ALL cell lines **A.** MTT assays of T-ALL and B-ALL cell lines treated for a total of 46 h with either one drug alone or with a combination (6 h of pre-treatment with bortezomib, followed by adding of CX-4945 for 40 h) of two drugs at a constant ratio of 1:2000 (bortezomib:CX-4945). The combined treatments resulted in a strong synergism (CIs 0.1-0.3), synergism (CIs 0.3-0.7), or moderate/slight synergism (CIs 0.7-0.9); data are representative of three independent experiments bars, s.d. CX, CX-4945; BZ, bortezomib. **B.** Flow cytometric analysis of Annexin V-FITC/PI-stained T-ALL and B-ALL cells treated for 24 h with CX-4945 (5 μM) and bortezomib (2.5 nM) either alone or in combination (6 h of pre-treatment with bortezomib, followed by adding of CX-4945 for 18 h). A significant increase in apoptotic cells is documented in samples treated with the drug combination with respect to single treatments. Asterisks indicate statistically significant differences (*P < 0.05; **P < 0.005; ***P < 0.0005). Results are the mean of three different experiments ±s.d. Ctrl, untreated cells; CX+BZ, drug combination.

We investigated whether CK2 and proteasome inhibition could cause synergistic effects in terms of reduced cell viability in ALL models. To this end, T- (MOLT-4, JURKAT, CEM-R) and B- (KOPN-8, RS4;11, NALM-6) ALL cell lines were incubated for 46 h total with either one drug alone or with a combination (6 h of pre-treatment with bortezomib, followed by adding of CX-4945 for 40 h) of two drugs at a constant ratio of 1:2000 (bortezomib:CX). Cell viability rates and C.I.s were then calculated. The combined treatment was strongly synergistic, as indicated by C.I.s well below 1, except for some CIs of CEM-R and NALM-6 cells (Figure 1A). Notably, synergistic effects were not observed after simultaneous combined treatment with bortezomib and CX-4945 (data not shown). To further evaluate whether the observed effects of bortezomib/CX-4945 treatment on cell viability could be related to apoptosis, flow cytometry analysis was performed. Cells were treated for 24 h with bortezomib (2.5 nM) and CX-4945 (5 μM) either alone or in combination (6 h of pre-treatment with bortezomib, followed by adding of CX-4945 for 18 h). We detected a statistically significant increase in the percentage of early apoptotic (single positive for Annexin V) and/or late apoptotic (double positive for Annexin V and PI) cells after drug combination treatment as compared with single treatments, especially in MOLT-4, KOPN-8 and RS4;11 cell lines (Figure 1B).

### Apoptosis induced by the bortezomib/CX-4945 combination has mitochondrial and ER-stress implications

**Figure 2:**
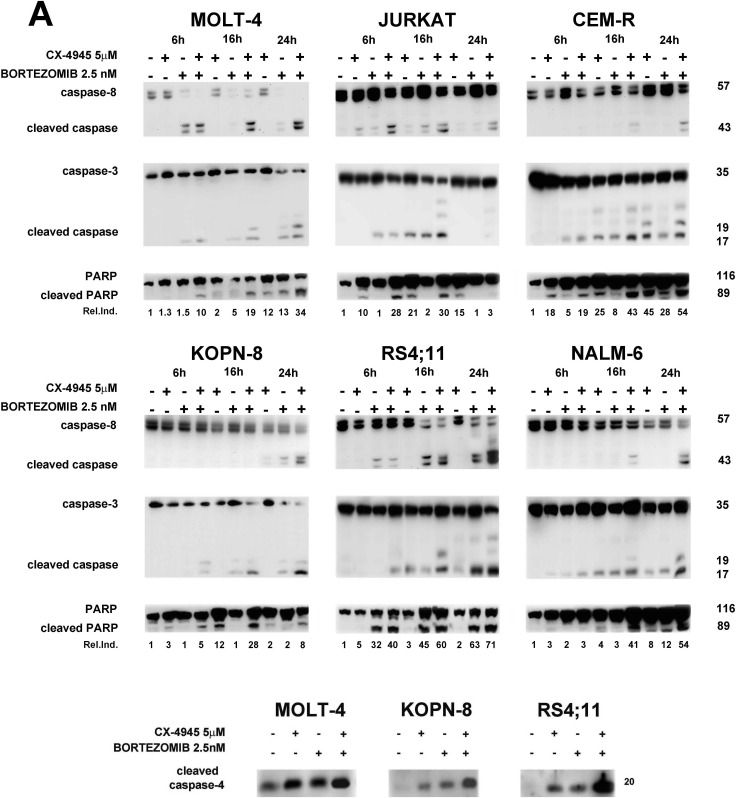

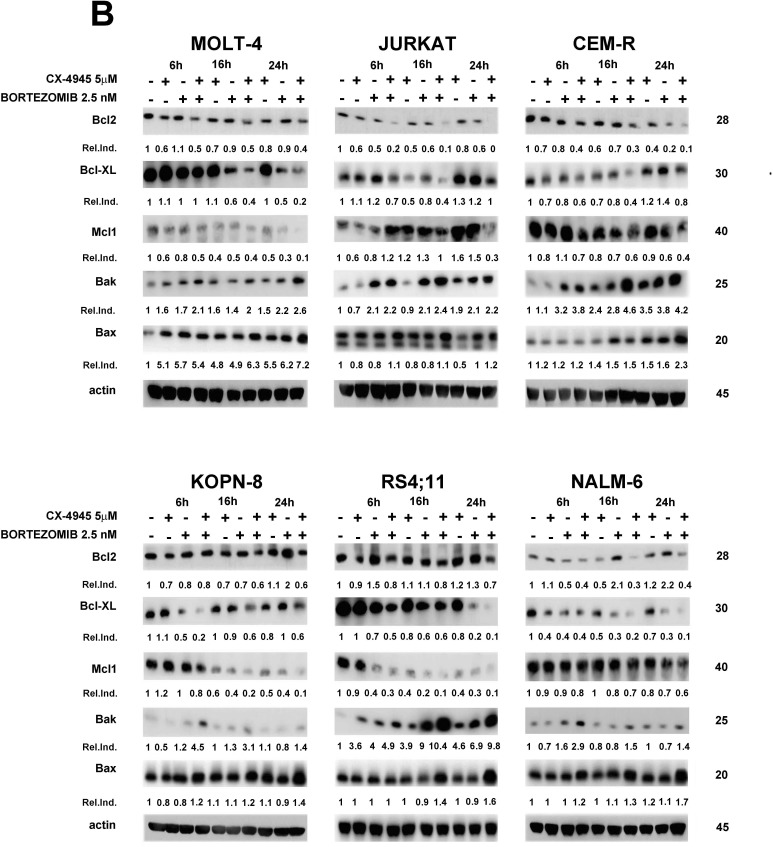
Apoptosis induced by the bortezomib/CX-4945 combination involves both mitochondrial and ER-stress **A.** Western blot analysis documenting a time-dependent cleavage of caspase-8, caspase-3, PARP and caspase-4. Densitometry scanning of cleaved PARP bands was performed. β-actin bands are not shown here, but are shown in B. **B.** Time-dependent modulation of Bcl-2 family members expression by CX-4945 (5 μM) and bortezomib (2.5 nM) either alone or in combination (6 h of pre-treatment with bortezomib, followed by adding of CX-4945 for 6, 16 and 24 h). Fifty μg of protein was blotted to each lane. Antibody to β-actin served as a loading control. Molecular weights are indicated at right. The Relative Induction (Rel.Ind.) is the amount of protein present in treated samples relative to untreated cells after normalizing to β-actin density.

Apoptosis induction was further assessed by western blot analysis of caspase-8, caspase-3 and poly (ADP-ribose) polymerase (PARP) cleavage. The drug combination was able to induce a more significant time-dependent cleavage of the three proteins, compared to single agent treatment. The time of maximum cleavage was different, depending on the cell line (Figure 2A). Cleaved PARP was quantified using densitometry scanning and results were showed as Relative Induction values (Rel.Ind.), the amount of protein present in treated samples relative to untreated cells after normalizing to actin band density. Given the roles played by both bortezomib [23, 35] and CX-4945 [26, 32] in ER stress/UPR mechanisms, we also analyzed in MOLT-4, KOPN-8 and RS4;11 cells, caspase-4 cleavage, a known marker of ER stress [36]. After 16 h of treatment, the drug combination caused a more marked cleavage than single drugs (Figure 2A). Mitochondrial involvement was also investigated through western blot analysis of Bcl-2 family members expression. As shown in Figure 2B, bortezomib/CX-4945 combination caused a time-dependent reduction of anti-apoptotic Bcl-2, Bcl-XL and Mcl1 as compared with single treatments, to a different extent depending on the cell line. In particular, in T-ALL cells and in NALM-6 cells, Bcl-2 down-modulation occurred already after 6 h of treatment, while in KOPN-8 and RS4;11 the decrease was detected after 24 h of treatment. Bcl-XL decreased already after 6 h in all cell lines. The same occurred for Mcl1 expression, except in JURKAT cells where, after 6 h of combined treatment, Mcl1 level increased and then decreased at 16 and 24 h, as confirmed by Rel.Ind. values. Pro-apoptotic Bax accumulation could be observed after 24 h of combined treatment, while Bak increase occurred earlier, already after 6 h of treatment. To better analyze mitochondrial involvement, we studied mitochondrial membrane potential by flow cytometry analysis of JC-1 dye [37] in RS4;11 cell line. As shown in Supplementary Figure S1, after 16 h of treatment, the drug combination was able to diminish red JC-1 fluorescence more than single treatments. Taken together, our findings indicated that bortezomib and CX-4945 cooperated to induce apoptotic cell death in ALL cells. Disrupted balance of Bcl-2 family members, mitochondrial depolarization and caspase-4 cleavage suggested the involvement of both mitochondrial and ER-stress mechanisms, respectively. The cleavage of caspase-8 could suggest the involvement of death receptor mechanism, therefore this pathway needs to be studied in more depth.

### Bortezomib/CX-4945 combination affects ER stress/UPR signaling

**Figure 3:**
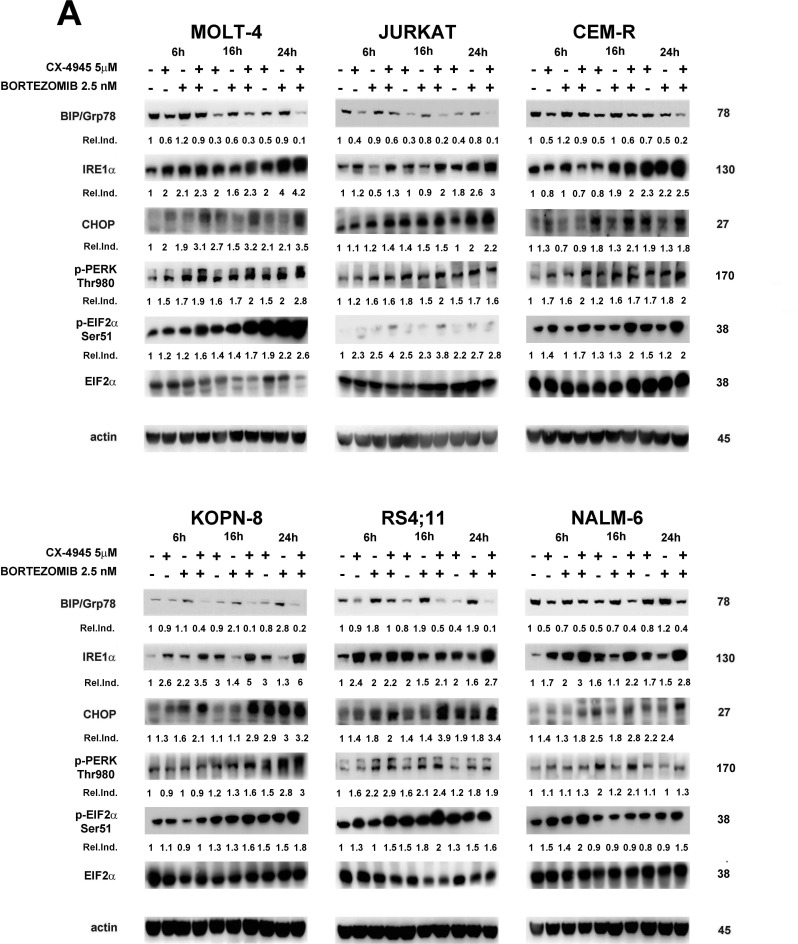

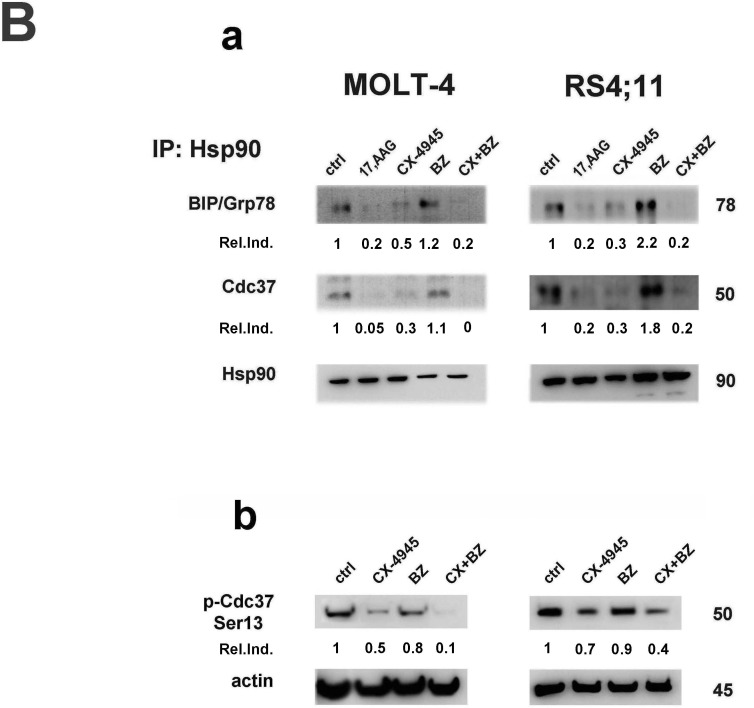
Bortezomib/CX-4945 combination modulates ER stress/UPR signaling in T- and B-ALL cell lines BIP/Grp78 depletion is provoked by impairment of Hsp90 chaperoning activity **A.** Western blot analysis documenting the time-dependent modulation of ER stress/UPR signaling markers induced by CX-4945 (5 μM) and bortezomib (2.5 nM) either alone or in combination (6 h of pre-treatment with bortezomib, followed by adding of CX-4945 for 6, 16 and 24 h). Fifty μg of protein was blotted to each lane. Antibody to β-actin served as a loading control. Molecular weights are indicated at right. Ser51 p-EIF2α Relative Induction values (Rel.Ind.) were obtained by normalizing to EIF2α density. **Ba**. Western blot analysis of immunoprecipitated Hsp90/BIP complexes in MOLT-4 (T-ALL) and RS4;11 (B-ALL) treated for 22 h with CX-4945 (5 μM) and bortezomib (2.5 nM) either alone or in combination (6 h of pre-treatment with bortezomib, followed by adding of CX-4945 for 16 h). 500 μg of protein lysates were immunoprecipitated with anti-Hsp90 antibody and then analyzed with the indicated antibodies. Relative Induction values (Rel.Ind.) were obtained by normalizing to Hsp90 bands density. **Bb**. Western blot analysis of Ser13 p-Cdc37 expression in MOLT-4 and RS4;11 cells treated for 22 h as previously described. Fifty μg of protein was blotted to each lane. Antibody to β-actin served as a loading control. Molecular weights are indicated at right. Ctrl, untreated cells; 17-AAG, tanespimycin; BZ, bortezomib; CX+BZ, drugs combination.

It is emerging that down-regulation of UPR signaling pathway drives apoptotic cell death also in pre-clinical settings of T-ALL [24, 26]. In contrast, bortezomib, causing ER lumen overload, is known to potently induce ER stress response [23, 36]. To better understand the molecular determinants of apoptosis induced by such opposing stimuli in ALL cell lines, we studied by western blot analysis the effects of bortezomib/CX-4945 combined treatment on ER stress/UPR markers expression. Regarding BIP/Grp78, a well-known mediator of the UPR pro-survival arm [38], we found that CX-4945 caused a time-dependent decrease of protein expression in all cell lines, whereas bortezomib rather induced an increase in almost all cell lines (MOLT-4 and CEM-R at 6 h, KOPN-8 and RS4;11 at all times of treatment, NALM-6 at 24 h) (Figure 3A). Intriguingly in combination with bortezomib, CX-4945 was able to overcome the opposite effect of the proteasome inhibitor, that reportedly is strongly correlated to resistance to bortezomib [18], leading to a stronger and more lasting BIP/Grp78 decrease. Densitometry analysis confirmed these observations (Figure 3A). In contrast, the expression of IRE1α, CHOP, Thr980 p-PERK and Ser51 p-EIF2α increased, to a different extent depending on the cell line, after exposure to CX-4945 and bortezomib as single treatments. This indicated for both drugs a concomitant induction of a significant stress in the ER lumen, as IRE1α, CHOP, p-PERK and p-EIF2α are well-established markers of ER stress/UPR-mediated apoptosis [39]. However, the combined treatment was able to induce an enhancement of the levels of these markers more markedly than single drugs, within the 24 h of treatment (Figure 3A). Therefore, our data suggested that sequential adding of CX-4945 after a previous exposure to bortezomib, could prevent ALL cells from buffering a stressed, overloaded ER lumen with a functional UPR, thus leading cells to apoptotic death more efficiently as compared with single agents. It has been reported [40] in MCL patient cells and in a mouse model of MCL xenotransplantation, that BIP/Grp78 depletion *via* its dissociation from Hsp90/BIP complexes led to inhibition of UPR and to apoptosis. Moreover, it is well known that CK2 inhibition, through dephosphorylation of Ser13 p-Cdc37, a Hsp90 co-chaperone, is able to impair Hsp90 activity [33]. To understand if BIP/Grp78 depletion, observed after the bortezomib/CX-4945 combined treatment, could be related to an impairment of the BIP/Hsp90/Cdc37 complex, we immunoprecipitated Hsp90 and we analyzed, by western blot, its association with BIP/Grp78 and Cdc37. In both MOLT-4 and RS4;11 cells, the combined treatment inhibited the interactions between Hsp90 and BIP/Grp78 more effectively than CX-4945 single treatment and to the same extent as tanespimycin (17-AAG), a specific Hsp90 inhibitor (Figure 3B). In contrast, bortezomib alone increased the association between Hsp90 and BIP/Grp78, as previously reported [18]. The levels of Cdc37 immunoprecipitated with Hsp90 followed the same trend of BIP/Grp78. Finally, western blot analysis of Ser13 p-Cdc37 levels confirmed that CX-4945, alone and in combination with bortezomib, through inhibition of Cdc37 phosphorylation, may drive the impairment of Hsp90 activity, leading to Hsp90/BIP dissociation, BIP/Grp78 depletion, and consequent inhibition of UPR. Densitometry analysis gives a quantitative support to these observations.

### A bortezomib/CX-4945 combined treatment affects STAT3 phosphorylation and NF-κB activation

**Figure 4:**
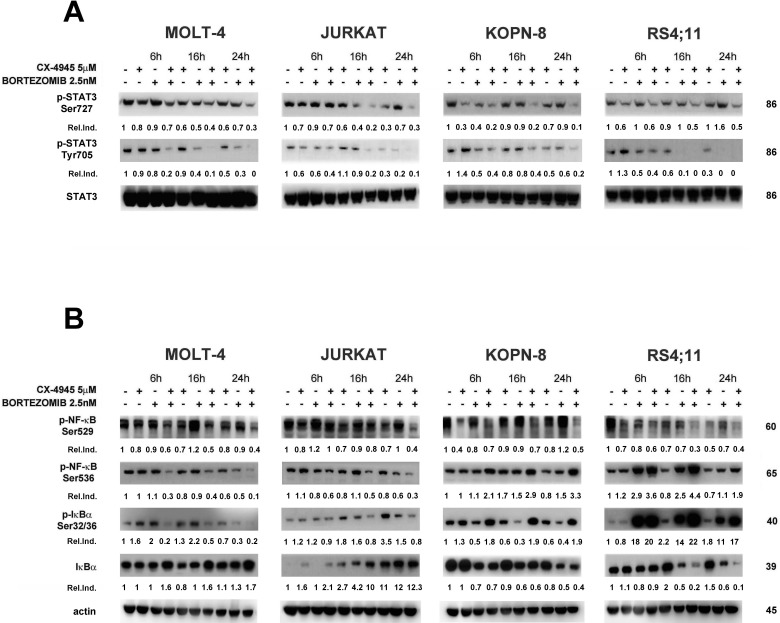

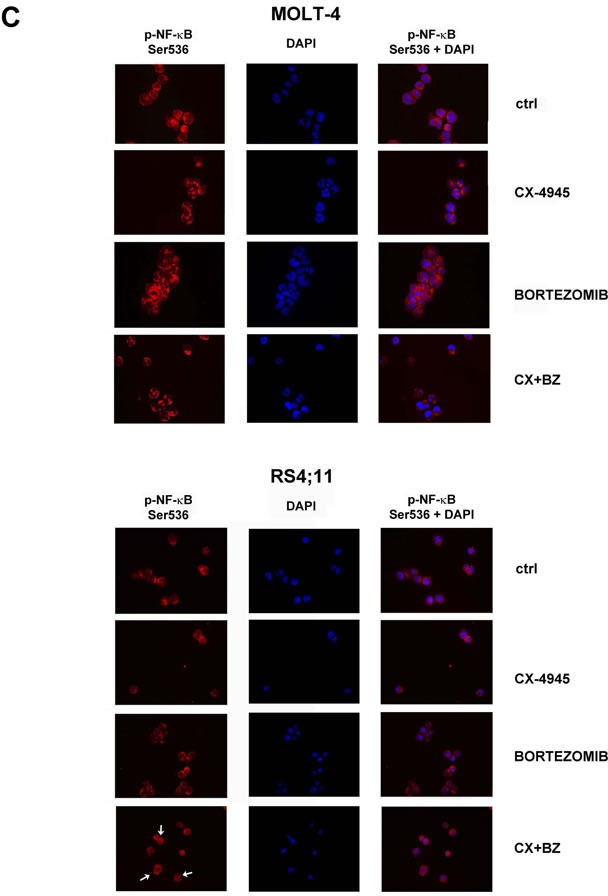
Effects of bortezomib/CX-4945 combined treatment on STAT3 phosphorylation and NF-κB activation **A.** Western blot analysis documenting the time-dependent dephosphorylation of Ser727 and Tyr705 p-STAT3 induced by CX-4945 (5 μM) and bortezomib (2.5 nM) either alone or in combination (6 h of pre-treatment with bortezomib, followed by adding of CX-4945 for 6, 16 and 24 h). Fifty μg of protein was blotted to each lane. Molecular weights are indicated at right. Relative Induction values (Rel.Ind.) were obtained by normalizing to STAT-3 density. **B.** Western blot analysis documenting the time-dependent modulation of NF-κB signaling after CX-4945 (5 μM) and bortezomib (2.5 nM) treatment. Cells were treated as previously described and Ser529 and Ser536 p-NF-κB, Ser32/36 p-IκBα, and IκBα levels were analyzed. The drug combination induced inhibition of the NF-κB axis in T-ALL cell lines (MOLT-4 and JURKAT) whereas NF-κB signaling was up-regulated in B-ALL cells (KOPN-8 and RS4;11). Molecular weights are indicated at right. **C.** Immunofluorescence analysis for Ser536 p-NF-κB cellular localization of MOLT-4 and RS4;11cells treated with CX-4945 (5 μM) and bortezomib (2.5 nM) either alone or in combination. Cells were treated, collected at 22 h and reacted with an antibody to Ser536 p-NF-κB which was revealed by a Cy3-conjugated secondary antibody. In RS4;11 samples combined treatment induces Ser536 p-NF-κB nuclear translocation (see white arrows). DAPI (4,6′-diamidino-2-phenylindole, dihydrochloride) was used to label nuclei. Ctrl, untreated cells; CX+BZ, drugs combination.

Both CK2 and proteasome inhibitions result in inactivation of the STAT3 and NF-κB survival pathways [41]. We analyzed the effects of CX-4945, bortezomib, and the combination of the two drugs on STAT3 and NF-κB p65 phosphorylation in ALL cell lines. Western blot analysis documented that CK2 inhibition by CX-4945 caused a reduction in Ser727 p-STAT3 (a known target of CK2 dependent phosphorylation) and also in Tyr705 p-STAT3 levels and this inhibition was potentiated after the exposure to the drug combination (Figure 4A). This finding correlates with previous observations obtained in MM and MCL preclinical settings [34]. Levels of Ser529 p-NF-κB, another known target site of CK2-dependent phosphorylation, decreased, as expected, in a time-dependent manner after CX-4945 exposure. Bortezomib increased phosphorylation at this residue (except for RS4;11 cells) but, intriguingly, the combined treatment within 24 h down-regulated Ser529 p-NF-κB more efficiently than CX-4945 alone, in all ALL cell lines (Figure 4B). Ser536 p-NF-κB levels displayed a different trend. In T-ALL cell lines (MOLT-4 and JURKAT) we observed a reduction of Ser536 phosphorylation after the combined treatment, with respect to single agents, while in B-ALL cell lines (KOPN-8 and RS4;11), Ser536 p-NF-κB was markedly induced by the bortezomib/CX-4945 combination (Figure 4B). Moreover, in T-ALL cell lines dephosphorylation of Ser536 p-NF-κB correlated with dephosphorylation of its inhibitor IκBα at Ser32/36 and with the increase in total levels of IκBα. Also in B-ALL cell lines, western blot analysis demonstrated a correlation between the increase in Ser536 p-NF-κB, induction of Ser32/36 p-IκBα, and reduction of total levels of IκBα. These differences in NF-κB phosphorylation were confirmed by immunofluorescence analysis of Ser536 p-NF-κB nuclear localization. Figure 4C shows that, in MOLT-4 cells, the bortezomib/CX-4945 combination did not induce Ser536 p-NF-κB nuclear translocation, whereas in RS4;11 cells the combined treatment caused a nuclear accumulation of Ser536 p-NF-κB. Taken together, these results indicate that in T-ALL cell lines the bortezomib/CX-4945 combination induced an inhibition of the NF-κB pathway, whereas in B-ALL cells, the combined treatment triggered NF-κB signaling activation.

### Bortezomib/CX-4945 combined treatment induces NF-κB pro-apoptotic activation in the RS4;11 cell line

**Figure 5:**
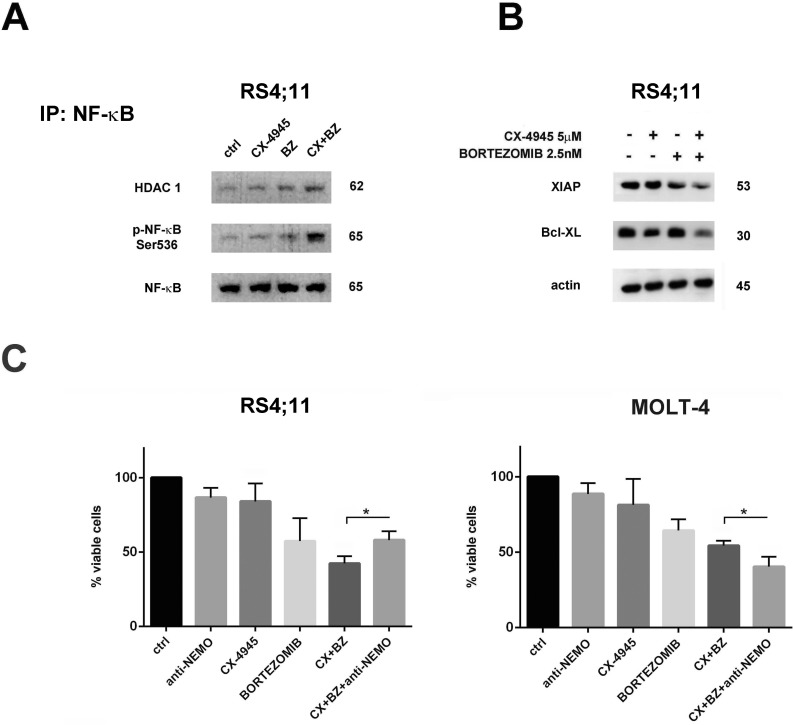
Bortezomib/CX-4945 combined treatment activated NF-κB pro-apoptotic functions in the RS4;11 cell line **A.** Western blot analysis of immunoprecipitated Ser536 p-NF-κB/HDAC1 complexes in RS4;11 treated for 22 h with CX-4945 (5 μM) and bortezomib (2.5 nM) either alone or in combination, as previously described. 500 μg of protein lysates were immunoprecipitated with anti NF-κB antibody and then analyzed with the indicated antibodies. **B.** Western blot analysis documenting the inhibition of XIAP and Bcl-xL expression levels induced by a 22 h treatment with CX-4945 (5 μM) and bortezomib (2.5 nM) either alone or in combination. Antibody to β-actin served as a loading control. Molecular weights are indicated at right. Ctrl, untreated cells; BZ, bortezomib; CX+BZ, drugs combination. **C.** Viable cell count assay of RS4;11 and MOLT-4 cell lines treated for 16 h with CX-4945 (5 μM) and bortezomib (2.5 nM) either alone or in combination (6 h of pre-treatment with bortezomib, followed by adding of CX-4945 for 10 h) and treated with inhibitor peptide of NEMO (IKKγ)-binding domain (anti-NEMO) either alone (for 4 h) or in combination with bortezomib/CX-4945 association. In RS4;11 cells, anti-NEMO peptide increased the number of viable cells, whereas in MOLT-4, it contributed to enhance the cytotoxic effects of the drug combination. Asterisks indicate statistically significant differences (*P < 0.05) between bortezomib/CX-4945 and bortezomib/CX-4945/anti-NEMO treatments. Ctrl, untreated cells; BZ, bortezomib; CX+BZ, drugs combination; anti-NEMO, inhibitor peptide of NEMO (IKKγ)-binding domain.

NF-κB activation observed in RS4;11 cells does not correlate with a lower sensitivity of this cell line to the bortezomib/CX-4945 combination, as indicated by MTT assays and apoptosis analysis. NF-κB is generally considered as an oncogenic and anti-apoptotic protein, however a growing body of evidence suggests it could also mediate cell death, depending on the cell context and apoptotic stimulus type [42, 43]. NF-κB can display its pro-apoptotic ability when it associates with histone deacetylases (HDACs), as in this way NF-κB turns from a transcriptional activator of several anti-apoptotic genes (Bcl-xL, XIAP, A20) to a transcriptional repressor of such genes [44]. To better assess if NF-κB activation, observed in RS4;11 cells after bortezomib/CX-4945 combined treatment, was related to acquisition of pro-apoptotic functions, we immunoprecipitated NF-κB and we analyzed, by western blot, its association with HDAC1. Figure 5A documents that bortezomib treatment induced a small increase in Ser536 p-NF-κB/HDAC1 complex formation, but adding CX-4945 significantly enhanced their association. To verify that p-NF-κB/HDAC1 complex formation could result in repression of NF-κB anti-apoptotic target genes, we analyzed both XIAP and Bcl-xL protein expression by western blot. In combined treatment samples (16 h) the levels of both proteins decreased (Figure 5B). To further document the effective contribution of NF-κB activation to the cytotoxic effects of bortezomib/CX-4945 treatment in RS4;11 cells, we added to the drug combination the inhibitor peptide of NEMO (IKKγ)-binding domain (anti-NEMO). We next performed a viable cell count assay. Adding anti-NEMO to RS4;11 cells increased the number of viable cells, whereas in MOLT-4, NF-κB inhibition contributed to enhance the cytotoxic effects of the drug combination (Figure 5C). Taken together these results suggest that, in B-ALL cells, but not in T-ALL cells, the bortezomib/CX-4945 combination triggers NF-κB activation. This activation is in a pro-apoptotic direction, in fact inhibition of NF-κB dampened the cytotoxic effects of the combined treatment.

### Bortezomib/CX-4945 combined treatment is effective in T- and B- ALL blasts

**Figure 6:**
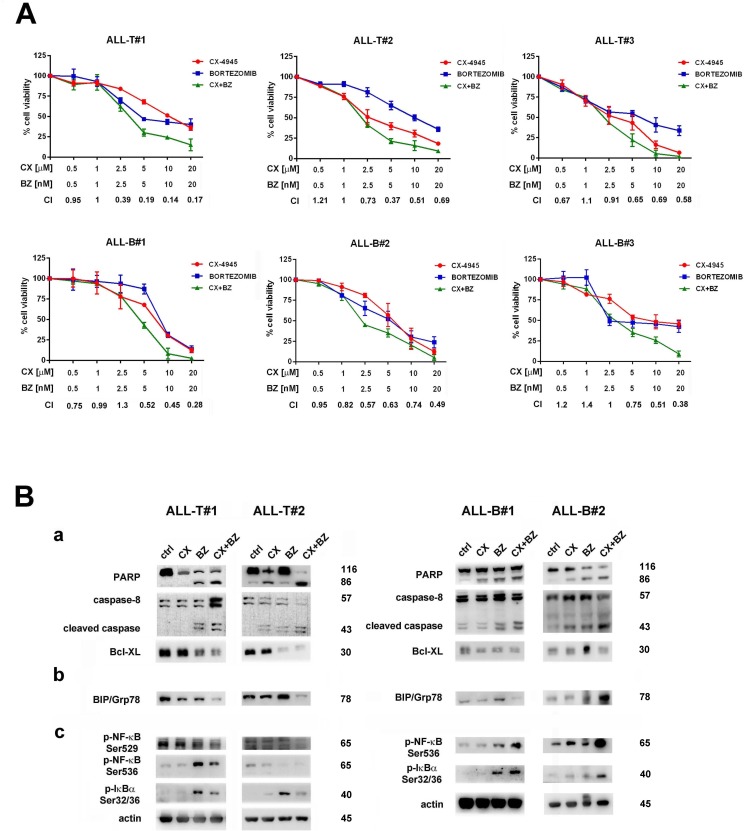
The Bortezomib/CX-4945 combined treatment is cytotoxic to primary lymphoblasts from T- and B-ALL patients, affects ER stress/UPR signaling and activates NF-κB pro-apoptotic functions **A.** MTT assays performed on primary cells from 3 pediatric T-ALL and 3 pediatric B-ALL patients treated for 46 h total with either one drug alone or with a combination (6 h of pre-treatment with bortezomib, followed by adding of CX-4945 for 40 h) of the two drugs at a constant ratio of 1:1000 (bortezomib:CX-4945). The combined treatment resulted in a strong synergism (CIs 0.1-0.3), synergism (CIs 0.3-0.7) or moderate/slight synergism (CIs 0.7-0.9); data are representative of three independent experiments, bars, s.d., CX, CX-4945; BZ, bortezomib. **B.** Western blot analysis of proteins extracted from two of T-ALL and two of B-ALL patient lymphoblasts, documenting **a.** the induction of apoptosis confirmed by cleavage of PARP, activation of caspase-8, and inhibition of Bcl-xL levels **b.** UPR inhibition, confirmed by BIP/Grp78 depletion, except for ALL-B#2 **c.** NF-κB pro-apoptotic activation, documented by induction of Ser536 p-NF-κB and Ser32/36 p-IκBα. Fifty μg of protein was blotted to each lane. Antibody to β-actin served as a loading control. Molecular weights are indicated at right. Ctrl, untreated cells; CX, CX-4945; BZ, bortezomib; CX+BZ, drugs combination.

To better assess the efficacy of the bortezomib/CX-4945 combined treatment as a potential therapeutic strategy in T- and B-ALL, we studied some pediatric T-ALL and B-ALL patient samples isolated from the bone marrow. Cells were incubated for a total of 46 h with either single drugs or a combination (6 h of pre-treatment with bortezomib, followed by adding CX-4945 for 40 h) of the two drugs at a constant ratio of 1:1000 (bortezomib:CX). Cell viability rates and C.I.s were calculated. The combined treatment displayed a good synergism, as indicated by the C.I.s (Figure 6A). Involvement of apoptosis, ER stress/UPR signaling, and NF-κB activation were assessed by western blot analysis after 16 h of treatment. Drug combination induced cleavage of both PARP and caspase-8 as well as a concomitant reduction of the proapoptotic Bcl-xL, both in T- and in B-ALL lymphoblasts (Figure 6B). Consistent with findings obtained in ALL cell lines, the combined treatment markedly down-regulated BIP/Grp78 protein levels, except for one B-ALL patient. In T-ALL patient lymphoblasts, the combined treatment down-modulated Ser529 p-NF-κB and was able to dampen the increase in Ser536 p-NF-κB, and Ser32/36 p-IκBα levels caused by bortezomib. Very interestingly, also in B-ALL primary cells, as observed in B-ALL cell lines, the drug combination induced Ser536 p-NF-κB and a concomitant p-IκBα increase (Figure 6B).

## DISCUSSION

Several lines of evidence have recently indicated that CK2 represents a pivotal molecule both regulating hematopoiesis-associated signaling cascades and driving the growth of different blood tumors. In fact CK2 plays a critical triggering role in several survival pathways including the PI3K/Akt/mTOR, WNT, NF-κB, and JAK-STAT networks, both in lymphoid and myeloid tumors [28, 45]. Preclinical evidence from studies testing the clinical-stage CK2 inhibitor CX-4945 in hematological malignancies, have demonstrated that there is a strong rationale for targeting CK2 in blood cancers and supported the initiation of clinical trials using CK2 antagonists. Previously, we provided evidence, in pre-clinical settings of T-ALL, that CX-4945 could induce apoptotic cell death through down-modulation of the UPR signaling pathway. Intriguingly, we have also demonstrated that a combination of CX-4945 with the ER stress inducer, thapsigargin, was synergistic in killing T-ALL cells [26]. ER stress and UPR pathways are activated in a wide variety of tumor types and have been demonstrated to be essential for tumor cell survival [46]. Although ER stress represents only one of the several processes associated with bortezomib-induced cell death [47], it is conceivable that it could indeed represent the key component [48, 49]. Acquisition of a more powerful UPR, due to deregulated expression of several heat shock protein family members, has been strongly associated with bortezomib resistance in a wide range of B-cell malignancies [38, 50, 51]. In the present study, we documented, in a panel of T- and B-ALL cell lines and primary samples, that bortezomib-dependent pro-apoptotic effects were significantly amplified by CX-4945 mediated inhibition of CK2. Apoptosis was accompanied by inhibition of UPR signaling, as evidenced by down-regulation of pro-survival ER chaperon BIP/Grp78. BIP/Grp78, when expressed at high levels, is involved in aggressive phenotype and chemoresistance in both proliferating and dormant cancer cells and it has been strongly correlated to resistance to bortezomib [40, 52-54]. We described that the combined treatment provoked BIP/Grp78 depletion, *via* decrease of the chaperoning activity of Hsp90. This is in agreement with findings obtained in MCL settings [40] and may explain the efficacy of heat shock protein inhibitors in combination with bortezomib [55-57]. Our observations indicated that dephosphorylation of co-chaperone Cdc37 at Ser13, which is CX-4945 mediated, seems to be the driving force for the impairment of BIP/Hsp90/Cdc37 complex, similarly to the findings of others for IRE1α [33]. Bortezomib/CX-4945 combination also induced ER stress more efficiently than single treatments, as demonstrated by up-regulation of IRE1α, CHOP, p-PERK and p-EIF2α expression levels. Thus, we hypothesized that the adding of CX-4945 after a previous bortezomib exposure, prevents leukemic cells to adequately cope with the stronger ER stress by properly activating UPR signaling. This pharmacological manipulation of ER stress is the base of the well documented efficacy of ER stress aggravators (ERSAs) (thapsigargin, tunicamycin, nelfinavir, etc) and has become an attractive approach for cancer therapeutic purposes [49, 51, 58]. We also demonstrated that the bortezomib/CX-4945 combination was able to trigger NF-κB activation in B-ALL models but not in T-ALL ones. In particular, in the RS4;11 cell line, we observed that this activation is in pro-apoptotic sense. The forming of the p-NF-κB/HDAC1 complex and the down-modulation of antiapoptotic XIAP and Bcl-xL levels may suggest the shift of NF-κB from a transcriptional activator of anti-apoptotic genes to a transcriptional repressor of them. We do not have direct evidence that reduced expression of these genes is the result of transcriptional repression by Ser536 p-NF-κB/HDAC1 complex, an issue that we are now investigating. Moreover, the reduction of cytotoxic effects of the combined treatment in RS4;11 cells after an anti-NEMO peptide treatment, further indicated that the drug combination was able to unleash NF-κB pro-apoptotic ability. It has been assumed that cancer cells activate NF-κB signaling in response to chemotherapy as a pro-survival strategy [59]. In contrast, a growing body of evidence indicates that NF-κB also has a tumor-suppressive function, which is heavily dependent on the stimuli and cell-type context [43, 60, 61]. It should be underlined that, despite its power to inhibit proteasome degradation of NF-κB inhibitor IκBα, bortezomib is already known to activate NF-κB signaling in ALLs and IκBα degradation has been described as a calpain-dependent event [62, 63]. Our observations indicated that CK2 inhibition could facilitate this bortezomib intrinsic ability, in B-ALL models. Very recently it has been discovered that phosphorylation of a highly conserved tyrosine residue, Tyr 57, in histone H2A was mediated by the unsuspected tyrosine kinase activity of CK2 and plays a critical role in transcriptional regulation. In fact, either inhibition of CK2 or Tyr 57 mutation impaired transcriptional elongation [64]. By western blotting, we analyzed phospho-tyrosine levels in histones isolated from RS4;11 cells treated with single drugs or with bortezomib/CX-4945 combination. We observed that the combined treatment was able to reduce phosphorylated tyrosine level in a protein band corresponding to histones H2A, more than single treatments (Supplementary Figure S2). This finding could explain the transcriptional repression of the anti-apoptotic genes normally activated by NF-κB in B-ALL models. At present, we do not have an explanation for the different behavior of NF-κB in T-ALL models. It has been demonstrated, in AML settings, that CX-4945 mediated CK2 inhibition caused a p53-dependent apoptosis [65]. T-ALL cell lines used in this study are p53 mutated, conversely B-ALL are p53 wild type but we did not observe a significant modulation of p53 active form after any drug treatments (data not shown).

Finally, we demonstrated a synergistic effect of bortezomib/CX-4945 combination also in primary cells from T- and B-ALL patients. Apoptotic cell death occurred through inhibition of UPR pathway and, as in the case of cell lines, while in B-ALL patients we observed NF-κB signaling activation, in T-ALL patients this did not occur. These findings are consistent with previous results describing a possible involvement of NF-κB as pro-apoptotic mediator in a panel of B-ALL cell lines, primary samples, and xenograft mouse models treated with a combination of bortezomib and HDAC inhibitors [22]. It is noteworthy that we have focused our observations on primary cells from a subset of immature B-ALL patients. However, when we tested the bortezomib/CX-4945 combination on a subset of mature B-ALL patients, in front of no NF-κB signaling activation, we have not observed any synergistic apoptotic effects (data not shown). Further experiments are necessary to establish if activation of NF-κB pro-apoptotic ability, *via* p-NF-κB/HDAC1 complex formation, plays a role in bortezomib/CX-4945 cytotoxicity in all B-ALL or only in some subsets of B-ALL.

In conclusion, in this work we provide evidence that inhibition of CK2 could enhance bortezomib-mediated cell death of ALL cells. Mechanisms involved in this synergism are multiple and each could represent an “Achilles heel” of cancer cell. Adding CX-4945 to bortezomib treatment resulted in UPR pathway inhibition through deletion of pro-survival ER chaperon BIP/Grp78 and, only in B-ALL models, the combination released the NF-κB pro-apoptotic ability. It is conceivable that the use of CK2 inhibitors in bortezomib-based combination protocols in ALL settings, could be a therapeutic opportunity, able to counteract the occurrence of bortezomib-resistant clones.

## MATERIALS AND METHODS

### Materials

Bortezomib, CX-4945, and tanespimycin (17-AAG) were kindly provided by Selleck Chemicals (Houston, TX, USA). Antibody to caspase-4 and Ser529 p-NF-κB were from Abcam (Cambridge, UK). Cdc37 antibody was from BD Biosciences (San Jose, CA, USA). All of the other primary and secondary antibodies for western blotting and flow cytometry analyses were from Cell Signaling Technology (Danvers, MA, USA). Cell Proliferation Kit I (MTT) was purchased from Roche Applied Science (Penzberg, Germany). NEMO (IKKγ)-binding domain inhibitor peptide was obtained from Imgenex (Novus Biologicals, Littleton, CO, USA).

### Cell culture and primary T-and B- ALL samples

T-ALL (MOLT-4, JURKAT, drug-resistant CCRF-CEM or CEM-R) and B-ALL (KOPN-8, RS4;11, NALM-6) cell lines were grown in RPMI 1640 medium, supplemented with either 10% or 20% heat-inactivated fetal bovine serum (FBS), depending on the cell line. Patient samples were obtained after informed consent according to Institutional guidelines and isolated by Ficoll-Paque (Amersham Biosciences AB, Uppsala, Sweden). T-ALL and B-ALL lymphoblasts were cultured in RPMI 1640 containing 20% FBS and ITS (insulin-transferrin-selenite).

### Cell viability analysis

MTT (3-[4,5-Dimethylthythiazol-2-yl]-2,5-Diphenyltetrazolium Bromide) assays were performed as previously reported [66]. For drug-combination experiments, a combination index (CI) number was calculated using the CalcuSyn software (BioSoft, Cambridge, UK) based on the Chou and Talalay method [67]. CI values between 0.1-0.9 define different grades of synergism: values between 0.9-1.1 are additive, whereas values > 1.1 are antagonistic.

### Annexin V-fluorescein isothiocyanate (FITC)/propidium iodide (PI) staining

To determine the extent of apoptosis induction after drug treatment, flow cytometric analysis of Annexin V-FITC/PI-stained samples was performed as previously reported [68]. All the flow cytometric analyses were performed on an FC500 flow cytometer (Beckman, Miami, FL, USA), with the appropriate software (CXP, Beckman).

### Flow cytometry analysis of JC-1 dye

Assessment of apoptosis induction was also obtained by detection of mitochondrial membrane potential using the 5,59,6,69, tetrachloro-1,1′,3,3′-tetraethylbenzimidazolyl carbocyanin iodide dye (JC-1) (Molecular Probes, Eugene, OR, USA) according to the manufacturer instructions.

### Western blot analysis and immunoprecipitation

Western blot analysis and immunoprecipitation were performed as previously detailed [69]. Cells were lysed using the M-PER Mammalian Protein Extraction Reagent, supplemented with the Protease and Phosphatase Inhibitor Cocktail (Thermo Fisher Scientific Inc., Rockford, IL, USA). BIP/Grp78 and HDAC immunoprecipitation was carried out using Pierce Classic IP Kit (Thermo Fisher Scientific Inc., Rockford, IL, USA), following the manufacturer instructions. Densitometry scanning of the bands were performed using a Chemidoc 810 Imager with the appropriate software (UVP, Upland, CA, USA).

### Histone isolation

Cells were lysed in PBS containing 0.5% Triton X100 (TEB) supplemented with the Protease and Phosphatase Inhibitor Cocktail (Thermo Fisher Scientific Inc., Rockford, IL, USA). After 10 min lysis on ice with gentle stirring, cells were centrifuged to spin down the nuclei. Nuclei pellet was washed in TEB and resuspended in 0.2 N HCl over night at 4°C to extract histones. After acid extraction, samples were centrifuged to remove debris. Supernatant containing histone proteins was mixed with acetone and incubated overnight at −20°C. The precipitated material was collected by microcentrifugation and air-dried. The acid-soluble histone fraction was dissolved in water and quantified for western blot analysis. Histones were immunoblotted with anti p-Tyr-100 antibody.

### Fluorescence immunocytochemistry

Fluorescence immunocytochemistry analysis were performed as previously described [70]. Cells were labeled with an antibody to Ser536 p-NF-κB, which was then detected by a Cy3-conjugated secondary antibody. DAPI (4,6′-diamidino-2-phenylindole, dihydrochloride) was used to counterstain nuclei. Images were taken under a Zeiss Axio Imager.Z1 microscope (Zeiss, Jena, Germany), with 40x/NA 0.75 optics, coupled to a computer-driven Zeiss AxioCAM digital camera (MRm), using the Zeiss AxioVision (version 4.5) software.

### Statistical analysis

The data are presented as the mean values from three separate experiments±s.d. Statistical analyses were performed using Student's *t* test at a significance level of *p* < 0.05 (GraphPad Prism Software).

## SUPPLEMENTARY MATERIAL FIGURES


