# Supplementary material for: Synergistic cytotoxic effects of bortezomib and CK2 inhibitor CX-4945 in acute lymphoblastic leukemia: turning off the prosurvival ER chaperone BIP/Grp78 and turning on the pro-apoptotic NF-κB
Source: Oncotarget. 2015 Nov 22;7(2):1323–40. doi: 10.18632/oncotarget.6361 (PMC4811463; doi:10.18632/oncotarget.6361)
Supplement: Supplementary file 1 [file oncotarget-07-1323-s001.pdf]

Synergistic cytotoxic effects of bortezomib and CK2 inhibitor CX-4945 in acute lymphoblastic leukemia: turning off the prosurvival ER chaperone BIP/Grp78 and turning on the pro-apoptotic NF-κB

Supplementary Material

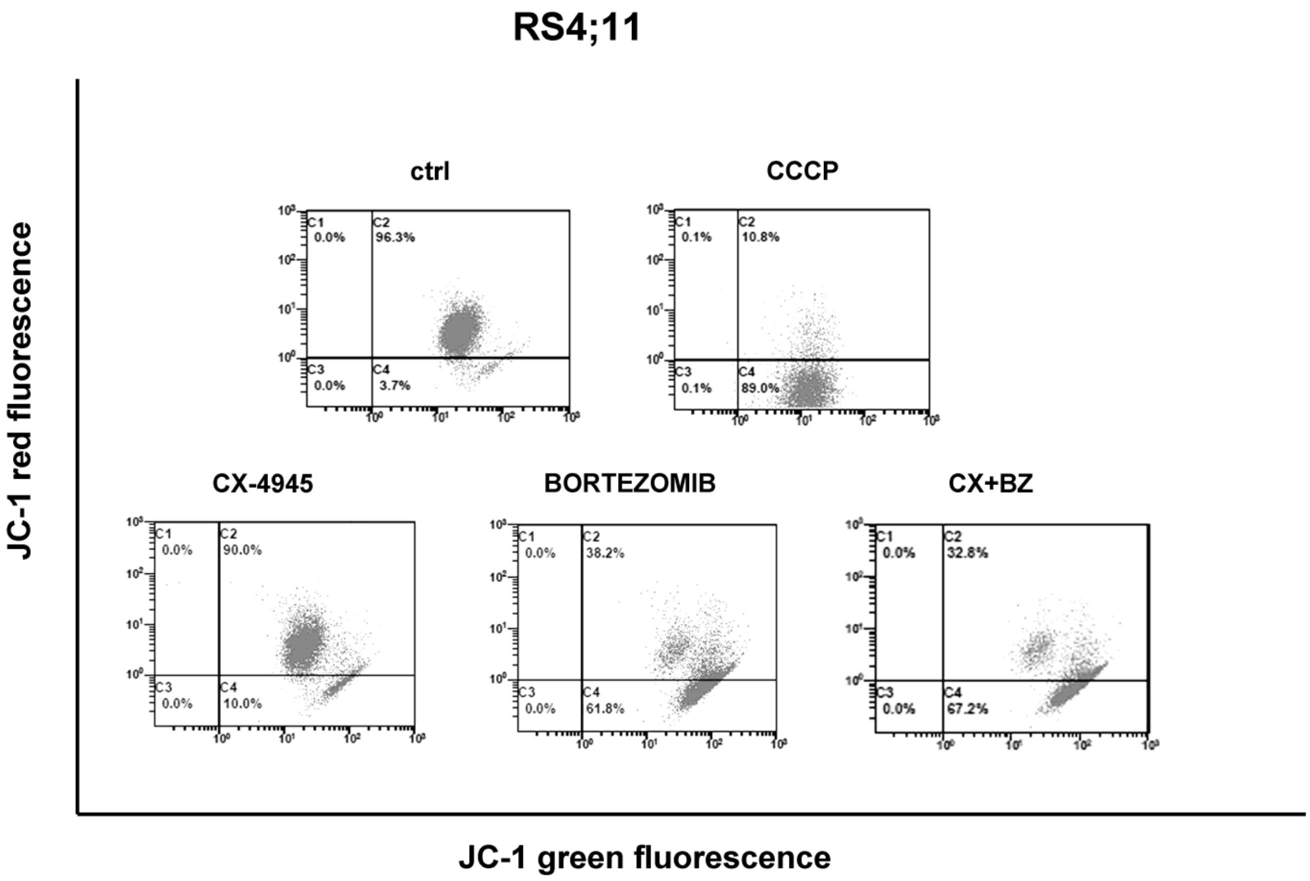

Supplementary Figure 1. Effects of bortezomib/CX-4945 combination on mitochondrial membrane potential. JC1 staining and flow cytometric analysis of RS4;11 cells treated with the mitochondrial membrane potential disrupter CCCP (carbonyl cyanide 3-chlorophenylhydrazone) used as positive control, with CX-4945 (5 μM) and bortezomib (2.5 nM) either alone or in combination. Ctrl, untreated cells; CX+BZ, drugs combination.

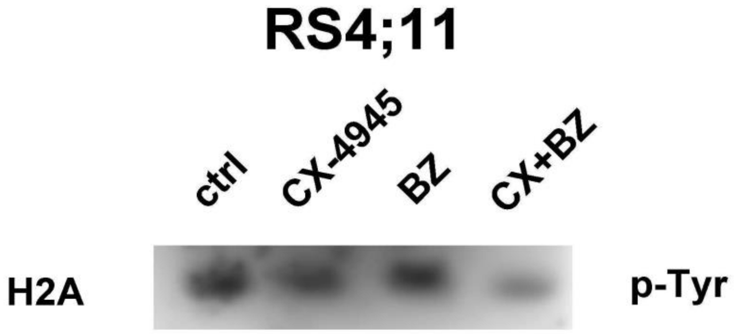

Supplementary Figure 2. Bortezomib/CX-4945 combined treatment reduces levels of p-Tyr histones H2A. Western blot analysis documenting phospho-tyrosine levels in histone H2A, isolated by acid extraction and electrophoretic separation, from RS4;11 cells treated for 22 h with CX-4945 (5 μM) and bortezomib (2.5 nM) either alone or in combination (6 h of pre-treatment with bortezomib, followed by adding of CX-4945 for 16 h). Ctrl, untreated cells; BZ, bortezomib; CX+BZ, drugs combination.
